# Super short operations on both gene order and intergenic sizes

**DOI:** 10.1186/s13015-019-0156-5

**Published:** 2019-11-05

**Authors:** Andre R. Oliveira, Géraldine Jean, Guillaume Fertin, Ulisses Dias, Zanoni Dias

**Affiliations:** 1 Institute of Computing, University of Campinas, Campinas, Brazil; 2 LS2N, UMR CNRS 6004, University of Nantes, Nantes, France; 3 School of Technology, University of Campinas, Limeira, Brazil

**Keywords:** Genome rearrangements, Intergenic regions, Super short operations, Approximation algorithms

## Abstract

**Background:**

The evolutionary distance between two genomes can be estimated by computing a minimum length sequence of operations, called *genome rearrangements*, that transform one genome into another. Usually, a genome is modeled as an ordered sequence of genes, and most of the studies in the genome rearrangement literature consist in shaping biological scenarios into mathematical models. For instance, allowing different genome rearrangements operations at the same time, adding constraints to these rearrangements (e.g., each rearrangement can affect at most a given number of genes), considering that a rearrangement implies a cost depending on its length rather than a unit cost, etc. Most of the works, however, have overlooked some important features inside genomes, such as the presence of sequences of nucleotides between genes, called *intergenic regions*.

**Results and conclusions:**

In this work, we investigate the problem of computing the distance between two genomes, taking into account both gene order and intergenic sizes. The genome rearrangement operations we consider here are constrained types of reversals and transpositions, called *super short reversals* (SSRs) and *super short transpositions* (SSTs), which affect up to two (consecutive) genes. We denote by *super short operations* (SSOs) any SSR or SST. We show 3-approximation algorithms when the orientation of the genes is not considered when we allow SSRs, SSTs, or SSOs, and 5-approximation algorithms when considering the orientation for either SSRs or SSOs. We also show that these algorithms improve their approximation factors when the input permutation has a higher number of inversions, where the approximation factor decreases from 3 to either 2 or 1.5, and from 5 to either 3 or 2.

## Background

Given two genomes ${\mathcal {G}}_1$ and ${\mathcal {G}}_2$, one way to estimate their evolutionary distance is to compute the minimum number of large scale events, called *genome rearrangements*, that are needed to transform ${\mathcal {G}}_1$ into ${\mathcal {G}}_2$. The minimality is required due to the commonly accepted parsimony principle, while the allowed genome rearrangements depend on the model, i.e. on the classes of events that supposedly happen during evolution.

Prior to counting rearrangement events, one needs to model the input genomes. Previous works [1–3] have defined genomes as ordered sequences of elements (*genes*). Variants within this setting can occur. For instance, each gene may appear either once or several times in a genome. In the latter case, genomes are modeled as strings, while in the former case they are modeled as *permutations*. Besides, genomes modeled as permutations may be signed or unsigned (the sign of an element represents the orientation of that gene in the DNA strand it lies on).

Concerning genome rearrangements, the most commonly studied events are *reversal*, which consists in taking a continuous sequence in the genome, reversing it, and putting it back at the same location [4], and *transposition*, which consists in taking a continuous sequence in the genome and putting it back in a different location [5]. A more recent and general type of genome rearrangement is the DCJ (Double-Cut and Join) [3], that cuts a genome between adjacent genes *a* and *b*, and adjacent genes *c* and *d*, and joins either *a* to *c* and *b* to *d*, or *a* to *d* and *b* to *c*.

Since the mid-nineties, a very large amount of work has been done for computing distances between pairs of genomes, depending on the genome model and the allowed set of rearrangements. We refer the reader to Fertin et al. book [6] for a survey of algorithmic aspects.

In populations where the number of rearrangement events that affect a very large portion of the genes are rare, we can restrict events to span at most *k* genes, for some value of *k* [7, 8]. During an analysis with closely-related pairs of bacterial genomes, the number of short inversions (inversions affecting up to three genes) was discovered to be very high, especially inversions of a single gene (which we call 1-reversal) [9]. There are also other works showing the prevalence of short inversions in bacterial genomes [10] and eukaryotes genomes  [11, 12].

As previously mentioned, most of the works have assumed that a genome is an ordered sequence of genes. It has been argued that this model could underestimate the “true” evolutionary distance, and that other genome features should be taken into account to circumvent this problem [13, 14]. Indeed, genomes carry more information than just their ordered sequences of genes. In particular, consecutive genes are separated by DNA sequences called *intergenic regions*, each having different lengths in terms of number of nucleotides. These lengths may be used along with gene order to generate a more realistic model for genomes.

This recently led some authors to model a genome as an ordered sequence of genes, together with an ordered list of its intergenic sizes, and to consider the problem of computing the DCJ distance, either in the case where insertions and deletions of nucleotides are forbidden [15], or allowed [16].

Biller and coauthors [13] used the intergenic regions to define what they called *fragile regions*, regions where rearrangements are more likely to act. After identifying these fragile regions, practical tests showed that considering rearrangements on non-fragile regions can yield incoherent distance estimations. When using the equiprobable model (i.e., rearrangements can occur in any position with the same probability), practical tests [16] showed that statistical properties of the inferred scenarios for DCJs using intergenic regions are closer to the true ones than scenarios which do not use them.

In this work, we also consider genomes as ordered sequences of genes together with their intergenic sizes, in cases where the gene sequence is an unsigned or signed permutation and the considered rearrangement operations are *super short reversal* (or SSR, i.e. a reversal of (gene) length at most two), *super short transposition* (or SST, i.e. a transposition affecting only two genes), or both (*super short operation* or SSO). In this context, our goal is to determine the minimum number of SSRs/SSTs/SSOs that transform one genome into another.

This paper is organized as follows. In Section 2 we provide the notations that we will use throughout the paper, and we introduce novel ideas that will prove useful for studying the problem. In sections 3-7 we derive lower and upper bounds on the sought distance for five different variants, which help us design an approximation algorithm of constant factor for each of these five problems. Section 8 presents a practical analysis of the five algorithms on simulated instances. Section 9 concludes the paper.

## Definitions

We represent a genome ${\mathcal {G}}$ with *n* genes as an instance with (i) an *n*-tuple and (ii) $n+1$ intergenic regions. If there is no duplicated genes, the *n*-tuple is a (possibly signed) permutation $\pi = (\pi _1\pi _2 \cdots$ $\pi _{n-1} \pi _n)$, with $|\pi _i| \in \{1,2,...,(n{-1}),n\}$, for $1 \le i \le n$, and $|\pi _i| = |\pi _j|$ if, and only if, $i = j$. If gene orientation is known, each element from $\pi$ has a $+$ or − sign that indicates the gene orientation it represents, and we say that $\pi$ is a *signed permutation*; $\pi$ is an *unsigned permutation* otherwise.

We denote by $\iota$ the *identity permutation*, in which all elements are in ascending order and with positive signs. The *extended permutation* is obtained from $\pi$ by adding two new elements: $\pi _0 = 0$ and $\pi _{n{+1}} = (n{+1})$.

The intergenic region $r_i^\pi$ is located before element $\pi _i$ from the extended permutation $\pi$, for $1 \le i \le n+1$. We denote by $\ell (r_i^\pi )$ the *length* of intergenic region $r_i^\pi$, i.e., the number of nucleotides in $r_i^\pi$, with $\ell (r_i^\pi ) \in {\mathbb {N}}$ for $1 \le i \le n+1$. Let $r^\pi = (\ell (r_1^\pi ),...,\ell (r_{n+1}^\pi ))$. An *instance* here is then formed by $(\pi ,r^\pi )$.

A *reversal* $\rho (i,j,x,y)$ applied over an instance $(\pi ,r^\pi )$, with $1 \le i \le j \le n$, $0 \le x \le \ell (r_{i}^\pi )$, $0 \le y \le \ell (r_{j+1}^\pi )$, and $\{x,y\} \in {\mathbb {N}}$, is an operation that generates $(\pi ',r^{\pi '})$ by (i) reversing the order and the orientation of the elements in the subset of adjacent elements $\{\pi _i,...,\pi _j\}$; (ii) reversing the order of intergenic regions in the subset of adjacent intergenic regions $\{r_{i+1}^\pi ,...,r_{j}^\pi \}$ when $j > i+1$; (iii) *cutting* two intergenic regions: $r_{i}^\pi$ after *x* nucleotides and $r_{j+1}^\pi$ after *y* nucleotides such that $\ell (r_{i}^{\pi '}) = x + y$ and $\ell (r_{j+1}^{\pi '}) = (\ell (r_{i}^\pi ){-x})+(\ell (r_{j+1}^\pi ){-y}).$

A reversal $\rho (i,j,x,y)$ is also called a *g*-reversal, where $g = (j-i)+1$. A *super short reversal* is a 1-reversal or a 2-reversal, i.e. a reversal that affects only one or two elements from $\pi$.

A *transposition* $\tau (i,j,k,x,y,z)$ applied over an instance $(\pi ,r^\pi )$, with $1 \le i< j < k \le n+1$, $0 \le x \le \ell (r_{i}^\pi )$, $0 \le y \le \ell (r_{j}^\pi )$, $0 \le z \le \ell (r_{k}^\pi )$, and $\{x,y,z\} \in {\mathbb {N}}$, is an operation that generates $(\pi ',r^{\pi '})$ by (i) exchanging subsets of adjacent elements $\{\pi _i,...,\pi _{j-1}\}$ and $\{\pi _j,...,\pi _{k-1}\}$; (ii) moving subsets of adjacent intergenic regions $\{r_{j+1}^\pi ,...,r_{k-1}^\pi \}$ and $\{r_{i+1}^\pi ,...,r_{j-1}^\pi \}$ to start at positions $(i+1)$ and $(i+k-j+1)$, respectively; (iii) *cutting* three intergenic regions: $r_{i}^\pi$, $r_{j}^\pi$, and $r_{k}^\pi$ such that $\ell (r_{i}^{\pi '}) = x + \ell (r_{j}^{\pi }){-y}$, $\ell (r_{i+k-j}^{\pi '}) = \ell (r_{i}^{\pi }){-x}+z$, and $\ell (r_{k}^{\pi '}) = \ell (r_{k}^\pi ){-z}+y$.

A transposition $\tau (i,j,k,x,y,z)$ is called a *g*-transposition, where $g=k-i$, and we say that a *g*-transposition is *super short* if $g = 2$.

**Fig. 1:**
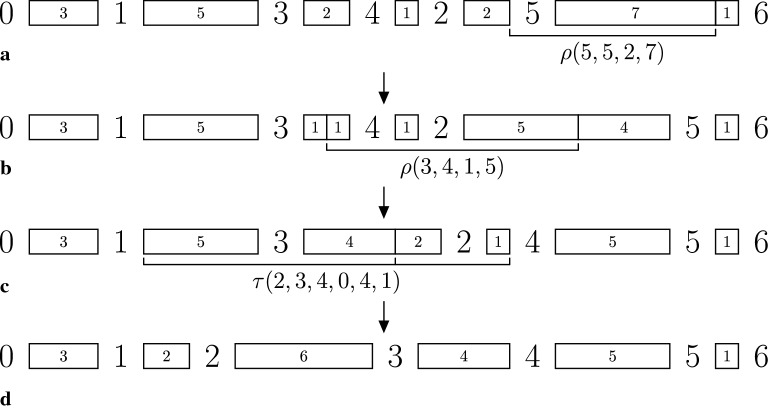
A sequence of two super short reversals and one super short transposition that transforms $\pi = (1~3~4~2~5)$, with $r^\pi = (3,5,2,1,2,8)$ into $\iota = (1,2,3,4,5)$, with $r^\iota = (3,2,6,4,5,1)$. Intergenic regions are represented by rectangles, whose dimensions vary according to their sizes. The 1-reversal $\rho (5,5,2,7)$ applied in **a** transforms $\pi$ into $\pi ' = \pi$, and it cuts $\pi$ after position 2 at $r^\pi _5$ and after position 7 at $r^\pi _6$, resulting in $\ell (r^{\pi '}_5) = 9$, $\ell (r^{\pi '}_6) = 1$, and $r^{\pi '} = (3,5,2,1,9,1)$. The 2-reversal $\rho (3,4,1,5)$ applied in **b** transforms $\pi '$ into $\pi '' = (1~3~2~4~5)$, and it cuts $\pi '$ after position 1 at $r^{\pi '}_3$ and after position 5 at $r^{\pi '}_5$, resulting in $\ell (r^{\pi ''}_3) = 6$, $\ell (r^{\pi ''}_5) = 5$, and $r^{\pi ''} = (3,5,6,1,5,1)$. Finally, the 2-transposition $\tau (2,3,4,0,4,1)$ applied in **c** transforms $\pi ''$ into $\iota$, and it cuts $\pi ''$ in position 0 at $r^{\pi ''}_2$, after position 4 at $r^{\pi ''}_3$, and after position 1 at $r^{\pi ''}_4$, resulting in $\ell (r^{\pi ''}_3) = 6$, $\ell (r^{\pi ''}_5) = 5$, and $r^{\pi ''} = (3,5,6,1,5,1)$. as shown in **d**

Figure 1 shows a sequence of two super short reversals and one super short transposition that transforms the permutation $\pi = (1\;3\;4\;2\;5)$ with $r^\pi = (3,5,2,1,2,8)$ into $\iota = (1\;2\;3\;4\;5)$ with $r^\iota = (3,2,6,4,5,1)$.

Given an instance $(\pi ,r^\pi )$, a pair of elements $(\pi _i,\pi _j)$ from $\pi$ is called an *inversion* if $\pi _i > \pi _j$ and $i < j$, with $\{i,j\} \in [1..n]$. We denote the number of inversions in a permutation $\pi$ by $inv(\pi )$. For the example in Fig. 1a, pairs (3, 2) and (4, 2) are the only inversions, thus $inv(\pi ) = 2$.

Given two instances $(\pi ,r^\pi )$ and $(\alpha ,r^\alpha )$ representing genomes ${\mathcal {G}}_1$ and ${\mathcal {G}}_2$ respectively such that $\pi$ and $\alpha$ have the same number of elements, $(\ell (r_i^\pi ) - \ell (r_i^\alpha ))$ is the *imbalance* between intergenic regions $r_i^\pi$ and $r_i^\alpha$, with $1 \le i \le m$.

Given two instances $(\pi ,r^\pi )$ and $(\alpha ,r^\alpha )$ such that (i) $\pi$ and $\alpha$ have the same number of elements and (ii) $\sum _{i=1}^m \ell (r_i^\pi ) = \sum _{i=1}^m \ell (r_i^\alpha )$, let $\Delta _j(r^\pi ,r^\alpha ) = \sum _{i=1}^{j} (\ell (r^\pi _i) - \ell (r^\alpha _i))$ denote the *cumulative sum of imbalances* between intergenic regions of $\pi$ and $\alpha$ from positions 1 to *j*, with $1 \le j \le m$. Since $\sum _{i=1}^m \ell (r_i^\pi ) = \sum _{i=1}^m \ell (r_i^\alpha )$, we have that $\Delta _m(r^\pi ,r^\alpha ) = 0$.

From now on, we will consider that (i) the target permutation $\alpha$ is such that $\alpha = \iota$; (ii) $\pi$ and $\iota$ have the same number of elements; and (iii) $\sum _{i=1}^m \ell (r_i^\pi ) = \sum _{i=1}^m \ell (r_i^\alpha )$. By doing this, we can compute the *distance* of $\pi$, denoted by $d(\pi )$, that consists in finding the minimum number of super short operations that sorts $\pi$ and transforms $r^\pi$ into $r^\iota$.

Let $(\pi ,r^\pi )$ and $(\iota ,r^\iota )$ be two instances such that $\pi$ and $\iota$ have the same number of elements and $\sum _{i=1}^m \ell (r_i^\pi ) = \sum _{i=1}^m \ell (r_i^\iota )$. The *intergenic graph*, denoted by $I(\pi ,r^\pi ,r^\iota ) = (V,E)$, is such that *V* is composed by two sets of vertices: intergenic vertices (one for each $r_i^\pi \in r^\pi$), and permutation vertices (one for each $\pi _i$ of the extended permutation $\pi$). The set *E* is composed by inversion edges: an edge $e = (r_i^\pi ,r_{i+2}^\pi ) \in E$ if there is a $j \ne i$ such that $(\pi _{i},\pi _{j})$ or $(\pi _{j},\pi _{i+1})$ is an inversion, with $1 \le i \le n{-1}$ and $1 \le j \le n$.

We divide vertices of an intergenic graph $I(\pi ,r^\pi ,r^\iota )$ into *components*. A component starts and ends with permutation vertices. Besides, the first component starts with the permutation vertex $\pi _0$, and the last component ends with the permutation vertex $\pi _{n{+1}}$. Consecutive components share exactly one permutation vertex, i.e., the last permutation vertex $\pi _i$ of a component is the first permutation vertex of its adjacent component to the right.

If a component *c* starts with vertex $\pi _i$ and ends with vertex $\pi _j$, with $i < j$, then $r_k^\pi \in c$ for $i < k \le j$ and $\pi _k \in c$ for $i< k < j$. Besides, any two intergenic vertices that are connected to each other by an inversion edge must belong to the same component. Thus, if *c* ends with $\pi _j$, then $e = (r^\pi _j,r^\pi _{j+2}) \not \in E$.

The idea is that components break $(\pi ,r^\pi )$ according to $I(\pi ,r^\pi ,r^\iota )$ into smaller pieces, where it is possible to make a local redistribution of intergenic regions and elements from $\pi$ (with no need to exchange them between components) transforming $(\pi ,r^\pi )$ into $(\iota ,r^\iota )$. This requires that any component *c* starting with $\pi _i$ and ending with $\pi _j$ must have $\sum _{k=i+1}^j \ell (r_k^\pi )-\ell (r_k^\iota ) = 0$.

Formally, given an intergenic graph $I(\pi ,r^\pi ,r^\iota )$, a *component* *c* is a minimal set of vertices from *V* in which: (i) any two intergenic vertices that are connected to each other by an inversion edge must belong to the same component, (ii) if $(r_i^\pi ,r_j^\pi ) \in g$, with $i<j$, then $\{\pi _{i-1},\pi _j\} \in c$ and for any $i< k < j$ $\{r_k^\pi ,\pi _k\} \in c$, and (iii) the sum of imbalances of its intergenic regions from $r^\pi$ with respect to $r^\iota$ is equal to zero, i.e. $\sum \nolimits _{\forall \, k \,s.t. \, r_k^\pi \in c} \ell (r_k^\pi )-\ell (r_k^\iota ) = 0$.

**Fig. 2:**
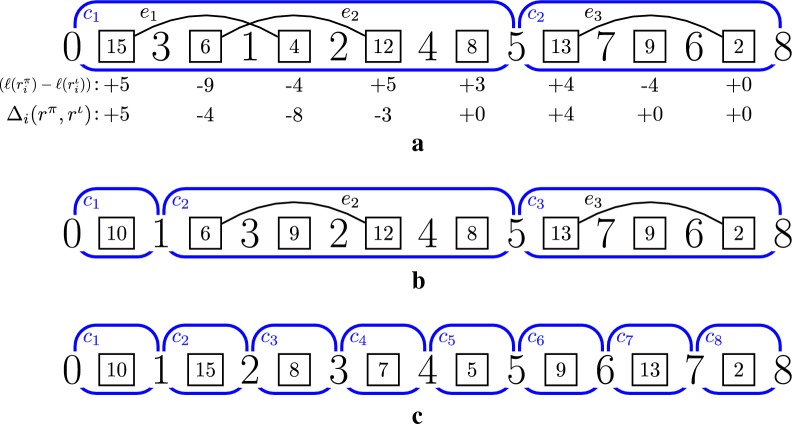
Intergenic graphs $I(\pi ,r^\pi ,r^\iota )$, $I({\pi '},r^{\pi '},r^\iota )$, and $I(\iota ,r^\iota ,r^\iota )$, with $\pi = (3~1~2~4~5~7~6)$, $r^\pi = (15,$ 6, 4, 12, 8, 13, 9, 2), $\pi ' = (1~3~2~4~5~7~6)$, $r^{\pi '} = (10,6,9,12,8,13,9,2)$, $\iota = (1~2~3~4~5~6~7)$, and $r^\iota = (10,15,8,7,5,9,13,2)$. Black squares represent intergenic vertices, and the number inside it indicate their sizes. Rounded rectangles in blue represent components. Note that in (**a**) there are three edges in $I(\pi ,r^\pi ,r^\iota )$, and $C(I(\pi ,r^\pi ,r^\iota )) = 2$, and $C_{odd}(I(\pi ,r^\pi ,r^\iota )) = 2$ since there are five intergenic vertices in $c_1$ and three intergenic vertices in $c_2$. We also have in (a) all values for $(\ell (r^\pi _i) - \ell (r^\iota _i))$ and $\Delta _i(r^\pi ,r^\iota )$, with $1 \le i \le 8$. The instance $(\pi ',r^{\pi '})$ is the result of applying $\rho (1,2,8,2)$ to $(\pi ,r^\pi )$. In (**b**) we can see that compared to $I(\pi ,r^\pi ,r^\iota )$, $I(\pi ',r^{\pi '},r^\iota )$ has one more component, and $e_1$ was removed. In (**c**) we can see that when we reach the target instance $(\iota ,r^\iota )$ the number of components is equal to the number of intergenic regions in $\iota$ (i.e., $C(I(\iota ,r^\iota ,r^\iota )) = n+1 = 8$)

A component with one intergenic vertex is called *trivial*, and is called *non-trivial* otherwise. The number of intergenic vertices in a component *c* is denoted by $c_r$. A component *c* is *odd* if $c_r$ is odd, and it is *even* otherwise. The number of components in an intergenic graph $I(\pi ,r^\pi ,r^\iota )$ is denoted by $C(I(\pi ,r^\pi ,r^\iota ))$, the number of odd components is denoted by $C_{odd}(I(\pi ,r^\pi ,r^\iota ))$, and the number of even components is denoted by $C_{even}(I(\pi ,r^\pi ,r^\iota ))$. Figure 2 shows three examples of intergenic graphs.

In the next two lemmas, we analyze the impact of applying super short operations on the number of components.

### **Lemma 1**

*Given an instance* $(\pi ,r^\pi )$ *and a target instance* $(\iota ,r^\iota )$, *let* $(\pi ',r^{\pi '})$ *be the resulting instance after applying a* 1*-reversal. It follows that* $C(I(\pi ',r^{\pi '},r^\iota )) \le C(I(\pi ,r^\pi ,r^\iota )) + 1$.

### *Proof*

Recall that a 1-reversal $\rho (i,i,x,y)$ is applied over intergenic regions $r_{i}^\pi$ and $r_{i+1}^\pi$, with $1 \le i \le n$. Besides, since 1-reversals do not create nor remove inversions from $\pi$, intergenic graphs $I(\pi ',r^{\pi '},r^\iota ) = (V',E')$ and $I(\pi ,r^{\pi },r^\iota ) = (V,E)$ satisfy $E = E'$.

**Fig. 3:**
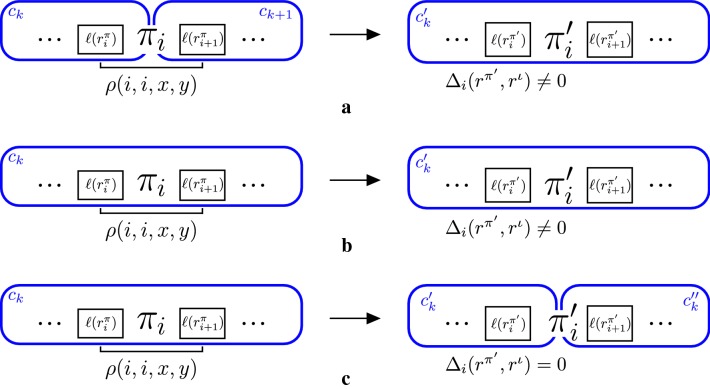
Example of intergenic graphs for all possible values of $C(I(\pi ',r^{\pi '},r^\iota ))$ with respect to $C(I(\pi ,r^\pi ,r^\iota ))$ where $(\pi ',r^{\pi '})$ is the resulting instance after applying a 1-reversal to $(\pi ,r^\pi )$. When the 1-reversal is applied over two components at the same time and $r^{\pi '}_i \ne r^\pi _i$, $C(I(\pi ',r^{\pi '},r^\iota )) = C(I(\pi ,r^\pi ,r^\iota ))-1$, as shown in **a**. Otherwise, we have that $C(I(\pi ',r^{\pi '},r^\iota )) = C(I(\pi ,r^\pi ,r^\iota )) + k$, $k \in \{0,1\}$, as shown in **b** and **c**

If $r_{i}^\pi \in c$ and $r_{i+1}^\pi \not \in c$, this 1-reversal is applied over two different components, which means that $r_{i}^\pi$ is the last intergenic region of *c*, so $\Delta _i(r^\pi ,r^\iota ) = 0$. If $x+y \ne \ell (r_i^\pi )$, we have that $C(I(\pi ',r^{\pi '},r^\iota )) = C(I(\pi ,r^{\pi },r^\iota )) -1$, as shown in Fig. 3a.

Consider now that $\{r_{i}^\pi ,r_{i+1}^\pi \} \in c$. If $i < n$ and $(r_i^{\pi '},r_{i+2}^{\pi '}) \in E'$, or $i > 1$ and $(r_{i-1}^{\pi '},r_{i+1}^{\pi '}) \in E'$, then $C(\pi ',r^{\pi '},r^\iota ) = C(\pi ,r^{\pi },r^\iota )$. Otherwise, we have two cases to consider: $C(\pi ',r^{\pi '},r^\iota ) = C(\pi ,r^{\pi },r^\iota )$, if $\Delta _i(r^{\pi '},r^\iota ) \ne 0$ (as shown in Fig. 3b); and $C(\pi ',r^{\pi '},r^\iota ) = C(\pi ,r^{\pi },r^\iota ) + 1$ if $\Delta _i(r^{\pi '},r^\iota ) = 0$ (as shown in Fig. 3c). $\square$

### **Lemma 2**

*Given an instance* $(\pi ,r^\pi )$ *and a target instance* $(\iota ,r^\iota )$, *let* $(\pi ',r^{\pi '})$ *be the resulting instance after applying either a* 2*-reversal or a* 2*-transposition. It follows that* $C(I(\pi ',r^{\pi '},r^\iota )) \le C(I(\pi ,r^\pi ,r^\iota )) + 2$.

### *Proof*

**Fig. 4:**
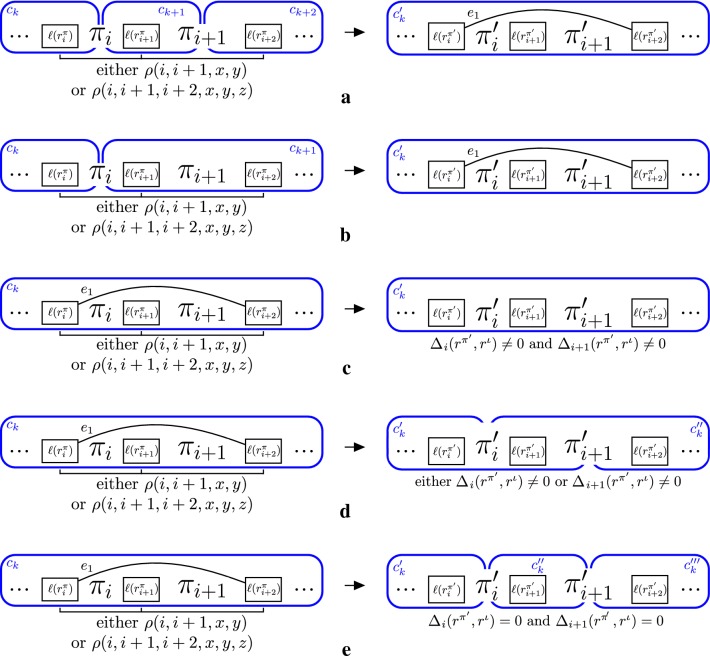
Example of intergenic graphs for all possible values of $C(I(\pi ',r^{\pi '},r^\iota ))$ with respect to $C(I(\pi ,r^{\pi },r^\iota ))$ where $(\pi ',r^{\pi '})$ is the resulting instance after applying a 2-reversal or a 2-transposition to $(\pi ,r^{\pi })$. When the 2-reversal or 2-transposition is applied over two components at the same time $C(I(\pi ',r^{\pi '},r^\iota )) < C(I(\pi ,r^{\pi },r^\iota ))$, as shown in **a** and **b**. Otherwise, we have that $C(I(\pi ,r^{\pi },r^\iota )) \le C(I(\pi ',r^{\pi '},r^\iota )) \le C(I(\pi ,r^{\pi },r^\iota )) + 2$, as shown in **c**–**e**

If a 2-reversal or 2-transposition is applied to intergenic regions of two different components in $I(\pi ,r^\pi ,r^\iota )$, then we are necessarily creating a new inversion, and the graph $I(\pi ',r^{\pi '},r^\iota )$ has either $C(I(\pi ',r^{\pi '},r^\iota )) = C(I(\pi ,r^{\pi },r^\iota )) -2$ (as shown in Fig. 4a) or $C(I(\pi ',r^{\pi '},r^\iota )) = C(I(\pi ,r^{\pi },r^\iota )) -1$ (as shown in Fig. 4b).

Consider now that this operation is applied to intergenic regions of a same component in $I(\pi ,r^\pi ,r^\iota )$, and exchanges elements $\pi _i$ and $\pi _{i+1}$, with $1 \le i < n-1$. If the intergenic graph $I(\pi ',r^{\pi '},r^\iota ) = (V',E')$ has $(r_{i}^{\pi '},r_{i+2}^{\pi '}) \in E'$, then $C(I(\pi ',r^{\pi '},r^\iota )) = C(I(\pi ,r^\pi ,r^\iota ))$. Otherwise, we have three cases to consider:

1. $C(I(\pi ',r^{\pi '},r^\iota )) = C(I(\pi ,r^{\pi },r^\iota ))$, if $\Delta _i(r^{\pi '},r^\iota ) \ne 0$ and $\Delta _{i+1}(r^{\pi '},r^\iota ) \ne 0$ (as shown in Fig. 4c);
2. $C(I(\pi ',r^{\pi '},r^\iota )) = C(I(\pi ,r^{\pi },r^\iota )) + 1$ if either $\Delta _i(r^{\pi '},r^\iota ) = 0$ or $\Delta _{i+1}(r^{\pi '},r^\iota ) = 0$ (as shown in Fig. 4d);
3. $C(I(\pi ',r^{\pi '},r^\iota )) = C(I(\pi ,r^{\pi },r^\iota )) + 2$ otherwise (as shown in Fig. 4e).$\square$

In the following sections, we will explore five different problems concerning super short operations but also considering intergenic regions, namely Sorting by Super Short Reversals (SbSSR), Sorting by Super Short Transpositions (SbSST), Sorting by Super Short Reversals and Super Short Transpositions (SbSSO), Sorting by Signed Super Short Reversals (SbSigSSR), and Sorting by Signed Super Short Reversals and Super Short Transpositions (SbSigSSO). Table 1 summarizes our results concerning general permutations (GP), permutations with $n\ell$ inversions for some $\ell \ge 1$ ($\ell$IP), permutations with at least *n* inversions (1IP), and permutations with at least 2*n* inversions (2IP).

**Table 1:** Summary of the approximation factor of the approximation algorithms presented in this manuscript for general permutations (GP), permutations with $n\ell$ inversions for some $\ell \ge 1$ ($\ell$IP), permutations with at least *n* inversions (1IP), and permutations with at least 2*n* inversions (2IP)

Sorting problem	GP	$\ell$IP	1IP	2IP
SbSSR	3	$1+\frac{1}{\ell }$	2	1.5
SbSST	3	$1+\frac{1}{\ell }$	2	1.5
SbSSO	3	$1+\frac{1}{\ell }$	2	1.5
SbSigSSR	5	$1+\frac{2}{\ell }$	3	2
SbSigSSO	5	$1+\frac{2}{\ell }$	3	2

## Sorting by Super Short Reversals

In this section, we analyze the version of the problem when only super short reversals (i.e., 1-reversals and 2-reversals) are allowed to transform $(\pi ,r^\pi )$ into $(\iota ,r^\iota )$. First, we state that if a non-trivial component *c* of an intergenic graph $I(\pi ,r^{\pi },r^\iota )$ has no edge (i.e., there is no inversion inside *c*), then it is always possible to split *c* into two components with a 1-reversal.

### **Lemma 3**

*If a component* *c* *of an intergenic graph* $I(\pi ,r^{\pi },r^\iota )$ *with* $c_r \ge 2$ *contains no edge, then there is always a pair of consecutive intergenic regions to which we can apply a* 1*-reversal that splits* *c* *into two components* $c'$ *and* $c''$ *such that* $c'_r+c''_r= c_r$.

### *Proof*

Let $p_i$ be the index in $r^\pi$ of the *i*-th intergenic region inside component *c*. The last intergenic region of *c* is at position $p_{c_r}$. By definition of a component, and since *c* contains no edge, for any $p_1 \le j < p_{c_r}$ we have that $\Delta _j(r^\pi ,r^\iota ) \ne 0$. Note that since $c_r > 1$ we have that $\Delta _{p_1}(r^\pi ,r^\iota ) = (\ell (r^\pi _{p_1}) - \ell (r^\iota _{p_1})) \ne 0$.

If $\Delta _{p_1}(r^\pi ,r^\iota ) > 0$, let $p_i = p_1$ and let *k* be the index of element from $\pi$ located right after $r_{p_1}^\pi$. Apply the reversal $\rho (k,k,\ell (r_{p_1}^\iota ),0)$.

Otherwise, we have that $\Delta _{p_1}(r^\pi ,r^\iota ) < 0$, and we need to find two intergenic regions $r_{p_i}^{\pi }$ and $r_{p_{i+1}}^{\pi }$ for $1 \le i < c_r$ such that $\Delta _{p_i}(r^\pi ,r^\iota ) < 0$ and $\Delta _{p_{i+1}}(r^\pi ,r^\iota ) \ge 0$. Since, by definition of a component, $\Delta _{p_{c_r}}(r^\pi ,r^\iota ) = 0$, such pair always exists. Let *k* be the index of element from $\pi$ located right after $r_{p_i}$. Apply the reversal $\rho (k,k,\ell (r_{p_i}^\pi ),-\Delta _{p_i}(r^\pi ,r^\iota ))$.

In both cases, the resulting permutation $\pi '$ has $\Delta _{p_i}(r^{\pi '},r^\iota ) = 0$, $\Delta _{p_{i+1}}(r^{\pi '},r^\iota ) = \Delta _{p_{i+1}}(r^\pi ,r^\iota )+\Delta _{p_i}(r^\pi ,r^\iota )$, and for any $i+2 \le j \le c_r$ we have that $\Delta _{p_j}(r^{\pi '},r^\iota ) = \Delta _{p_j}(r^\pi ,r^\iota )$ ; thus, as before, all intergenic regions from $r_{p_{i+1}}^{\pi '}$ to $r_{p_{c_r}}^{\pi '}$ must belong to the same component.

This 1-reversal splits *c* into two components: $c'$ with all intergenic regions in positions $p_1$ to $p_i$, and $c''$ with all intergenic regions in positions $p_{i{+1}}$ to $p_{c_r}$. $\square$

Let $\Delta _{odd}(r^\pi ,r^\iota ) = \sum _{i=1,\, i \pmod {2} = 1}^{n+1} (\ell (r^\pi _i) - \ell (r^\iota _i))$ denote the cumulative sum of imbalances of intergenic regions from $\pi$ and $\iota$ in odd positions only. Using Lemmas 1, 2 and 3, we show in the following two lemmas the minimum and maximum number of super short reversals needed to transform $\pi$ into $\iota$ and $r^\pi$ into $r^\iota$.

### **Lemma 4**

*Let* $(\pi ,r^\pi )$ *be an instance,* $(\iota ,r^\iota )$ *be the target instance,* *m* *the number of intergenic regions in* $r^\pi$ *and* $r^\iota$, *and let* $\varphi ^{r} = 0$ *if* $\Delta _{odd}(r^\pi ,r^\iota ) = 0$ *and* $\varphi ^{r} = 1$ *otherwise. It follows that* $d(\pi ) \ge \max \{\frac{m-C(I(\pi ,r^\pi ,r^\iota ))}{2},inv(\pi ) + \varphi ^{r}\}$.

### *Proof*

In order to sort $\pi$, we need to remove all inversions, and since a 2-reversal can remove only one inversion, we have that $d(\pi ) \ge inv(\pi )$. Besides, since 2-reversals exchange material between intergenic regions of same parity only, then $d(\pi ) \ge inv(\pi ) + \varphi ^{r}$, with $\varphi ^{r} = 1$ if $\Delta _{odd}(r^\pi ,r^\iota ) \ne 0$ (in this case we will need at least one 1-reversal to exchange material between an intergenic region located at an odd position and an intergenic region located at an even position), and $\varphi ^{r} = 0$ otherwise.

On the other hand, by Lemmas 1 and 2, we can increase the number of components by at most two with a super short reversal, so to reach *m* trivial components we need at least $\frac{m-C(I(\pi ,r^\pi ,r^\iota ))}{2}$ super short reversals. $\square$

### **Lemma 5**

*Let* $(\pi ,r^\pi )$ *be an instance,* $(\iota ,r^\iota )$ *be the target instance, and let* *m* *be the number of intergenic regions in* $r^\pi$ *and* $r^\iota$. *We have that* $d(\pi ) \le inv(\pi ) + m-C(I(\pi ,r^\pi ,r^\iota ))$.

### *Proof*

While $\pi \ne \iota$, $\pi$ has at least one pair of consecutive elements $(\pi _i,\pi _{i+1})$ that is an inversion. Suppose that we first remove all inversions from $\pi$ using $inv(\pi )$ 2-reversals of type $\rho (i,i+1,\ell (r_i^{\pi }),0)$ i.e., without modifying its intergenic regions lengths. Let $\pi '$ be the resulting permutation, that has $r^{\pi '} = r^\pi$. The number of components in $I(\pi ',r^{\pi '},r^\iota )$ cannot be smaller than $C(I(\pi ,r^\pi ,r^\iota ))$, since any 2-reversal removing an inversion is applied inside a same component. By Lemma 3, we can go from $C(I(\pi ',r^{\pi '},r^\iota ))$ to *m* components using $m-{C(I(\pi ',r^{\pi '},r^\iota ))}$ 1-reversals, which results in no more than $m{-C(I(\pi ,r^{\pi },r^\iota ))}$ 1-reversals. $\square$

Finally, using Lemmas 4 and 5, we prove that it is possible to obtain 3-approximation for this problem.

### **Theorem 1**

*Let* $(\pi ,r^\pi )$ *be an instance,* $(\iota ,r^\iota )$ *be the target instance, and let* $m=n+1$ *be the number of intergenic regions in* $r^\pi$ *and* $r^\iota$. *The value of* $d(\pi )$ *is* 3-*approximable.*

### *Proof*

Let $k = C(I(\pi ,r^\pi ,r^\iota ))$, and let $\varphi ^{r} = 0$, if $\Delta _{odd}(r^\pi ,r^\iota ) = 0$, or $\varphi ^{r} = 1$ otherwise. If $\frac{m-k}{2} \ge inv(\pi ) + \varphi ^{r}$ then, by Lemma 4, $d(\pi ) \ge \frac{m-k}{2}$, and, by Lemma 5, $d(\pi ) \le m-k+inv(\pi ) \le m-k +\frac{m-k}{2} \le 3 \frac{m-k}{2}$. Otherwise, $\frac{m-k}{2} < inv(\pi )+\varphi ^{r}$, so $m-k < 2inv(\pi )+2\varphi ^{r}$. By Lemma 4, $d(\pi ) \ge inv(\pi )+\varphi ^{r}$, and, by Lemma 5, $d(\pi ) \le m-k+inv(\pi ) \le 2 inv(\pi ) + 2\varphi ^{r} + inv(\pi ) \le 3inv(\pi )+2\varphi ^{r}$. $\square$

Algorithm 1 describes a 3-approximation algorithm that transforms an instance $(\pi ,r^\pi )$ into $(\iota ,r^\iota )$ using super short reversals. Computing $inv(\pi )$ takes $O(n\log {n})$ and it takes up to $O(n^2)$ to build $I(\pi ,r^\pi ,r^\iota )$. Computing $\Delta _i(r^\pi ,r^\iota )$ and $C(I(\pi ,r^\pi ,r^\iota ))$ take *O*(*n*), and it takes constant time to update them. The while loop in line 5 (resp. line 14) iterates up to $O(n^2)$ times, so the overall complexity of Algorithm 1 is $O(n^2)$.

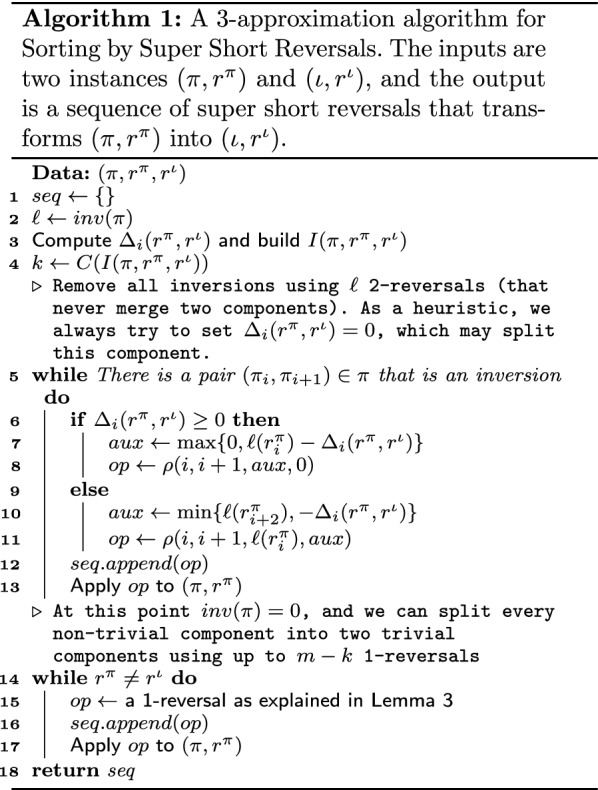



Let $\delta _n$ denote the set of all permutations $\pi$ with *n* elements, and let $\delta _{n,k}$ denote the number of all permutations $\pi \in \delta _n$ such that $inv(\pi ) \le k$. For $n = 12$ there are 762,007 permutations in $\delta _{12,12}$, which corresponds to 0.16% of the 12! permutations from $\delta _{12}$, and for $n > 12$ the number of permutations in $\delta _{n,n}$ never corresponds to more than 0.05% of the *n*! permutations from $\delta _n$ [17]. Besides, for $n > 18$ the number of permutations in $\delta _{n,2n}$ never exceeds 0.03% of the *n*! permutations from $\delta _n$ [17].

Algorithm 1 has a better approximation factor when the number of inversions is at least *n*, as explained in the following theorem.

### **Theorem 2**

*Let* $(\pi ,r^\pi )$ *be an instance,* $(\iota ,r^\iota )$ *be the target instance, and let* $m = n+1$ *be the number of intergenic regions in* $r^\pi$ *and* $r^\iota$. *If* $inv(\pi ) \ge n$, *Algorithm* 1 *has an approximation factor of* $(1+\frac{1}{\ell })$, *where* $\ell = \frac{inv(\pi )}{n} \ge 1$.

### *Proof*

Let $k = C(I(\pi ,r^\pi ,r^\iota ))$, and let $\varphi ^{r} = 0$ if $\Delta _{odd}(r^\pi ,r^\iota ) = 0$ and $\varphi ^{r} = 1$ otherwise. Suppose now that $inv(\pi ) = n\ell$ for some $\ell \ge 1$. Since $\frac{m-k}{2} < n$, by Lemma 4 we have that $d(\pi ) \ge n\ell$. Algorithm 1 applies $n\ell$ 2-reversals and up to $m-k < n$ 1-reversals, which results in no more than $n\ell + n-1 < n(\ell +1)$ super short reversals. $\square$

### Corollary 2.1

*Let* $(\pi ,r^\pi )$ *be an instance,* $(\iota ,r^\iota )$ *be the target instance, and let* $m = n+1$ *be the number of intergenic regions in* $r^\pi$ *and* $r^\iota$. *If* $inv(\pi ) \ge n$ (*resp.* $inv(\pi ) \ge 2n$) *Algorithm* 1 *has an approximation factor of at most 2 (resp. 1.5)*.

## Sorting by Super Short Transpositions

In this section, we analyze the version of the problem when only Super Short Transpositions are allowed. First, we investigate how 2-transpositions split non-trivial components from an intergenic graph $I(\pi ,r^\pi ,r^\iota )$.

### **Lemma 6**

*If a component* *c* *of an intergenic graph* $I(\pi ,r^\pi ,r^\iota )$ *with* $c_r > 2$ (*resp.* $c_r = 2$) *has no edge, then we can apply two* 2*-transpositions that split* *c* *into three components* $c'$, $c''$, *and* $c'''$ *such that* $c'_r+c''_r+c'''_r= c_r$ (*resp. two components* $c'$ *and* $c''$ *such that* $c'_r = c''_r = 1$).

### *Proof*

Note that any 2-transposition will increase or decrease the number of inversions by one. By Lemma 2, a 2-transposition that removes an inversion can increase the number of components by at most two units, and a 2-transposition creating an inversion cannot increase the number of components. Since there is no inversion in *c*, for each 2-transposition removing an inversion from *c* we have a 2-transposition creating that inversion before.

Now we explain how to increase the number of components by two units when $c_r \ge 3$. Let $p_i$ be the index in $r^\pi$ of the *i*-th intergenic region inside component *c*. If there is no intergenic region $r_j$ inside *c* in which the cumulative sum is negative, apply $\tau (p_1,p_2,p_3,x,y,0)$ in such a way that $x = \min \{\ell (r^\iota _{p_1}) + \ell (r^\iota _{p_2}), \ell (r^\pi _{p_1})\}$ and $y = \ell (r^\pi _{p_2}) + \ell (r^\iota _{p_1}) + \ell (r^\iota _{p_2}) - x$. Now apply $\tau (p_1,p_2,p_3,\ell (r^\iota _{p_1}),0,0)$. These two 2-transpositions split *c* into three components: $c'$ with $r_{p_1}$, $c''$ with $r_{p_2}$ and $c'''$ with the remaining intergenic regions from *c*. Note that $c'$ and $c''$ are odd, and $c'''$ has the same parity as *c*.

Otherwise, we can find a pair of consecutive intergenic regions $r_{p_i}$ and $r_{p_{i+1}}$ inside *c* such that $\Delta _{p_{i}}(r^\pi ,r^\iota ) < 0$ and $\Delta _{p_{i+1}}(r^\pi ,r^\iota ) \ge 0$, and since $\Delta _{p_{c_r}}(r^\pi ,r^\iota ) = 0$, such pair always exists. If $c_r$ is even or if $c_r$ is odd but $p_i$ is even, apply $\tau (p_{i-1},p_i,p_{i+1},x,y,$ 0) such that $x = \ell (r^\pi _{p_{i-1}})$ and $y = \ell (r^\pi _{p_{i}})+\Delta _{p{i-1}}(r^\pi ,r^\iota )$, followed by $\tau (p_{i-1},p_i,p_{i+1},x',0,y')$ such that $x' = \ell (r^\iota _{p_{i-1}})$ and $y'=\ell (r^\iota _{p_{i}})$.

If $c_r$ and $p_i$ are odd, apply $\tau (p_i,p_{i+1},p_{i+2},x,y,0)$ such that $x = \ell (r^\pi _{p_i})$ and $y = \ell (r^\pi _{p_{i+1}})+\Delta _{p{i}}(r^\pi ,r^\iota )$, followed by $\tau (p_{i-1},p_i,p_{i+1},x',0,y')$ such that $x' = \ell (r^\iota _{p_{i}})$ and $y' = \ell (r^\iota _{p_{i+1}})$. These two 2-transpositions split *c* into three components so if $c_r$ is even then we will end up with two odd components and one even component, and if *c* is odd we will end up with three odd components due to the choice of the position defined above.

If $c_r = 2$ and $p_1 > 1$ we apply $\tau (p_1-1,p_1,p_2,x,y,0)$ such that $x = \ell (r^\pi _{p_1-1})$ and $y = \ell (r^\pi _{p_1})$, followed by $\tau (p_1-1,p_1,p_2,x',0,y')$ such that $x' = x$ and $y' = \ell (r^\iota _{p_1})$. If $c_r = 2$ and $p_1 = 1$ we apply $\tau (p_1,p_2,p_2+1,\ell (r^\pi _{p_1}),0,0)$ followed by $\tau (p_1,p_2,p_2+1,\ell (r^\iota _{p_1}),0,0)$. These two transpositions transform *c* into two trivial components. $\square$

The following lemma gives the number of transpositions needed to transform a permutation $\pi$ and its intergenic regions $r^\pi$ into $\iota$ with its intergenic regions $r^\iota$ when $inv(\pi ) = 0$.

### **Lemma 7**

*Let* $(\pi ,r^\pi )$ *be an instance,* $(\iota ,r^\iota )$ *be the target instance, and let* $m=n+1$ *be the number of intergenic regions in* $r^\pi$ *and* $r^\iota$. *If* $inv(\pi ) = 0$, *then* $d(\pi ) = m - C(I(\pi ,r^\pi ,r^\iota )) + C_{even}(I(\pi ,r^\pi ,r^\iota ))$.

### *Proof*

If a 2-transposition applied on a component *c* of $I(\pi ,r^\pi ,r^\iota )$ increases the number of components by two units, we can assume by the proof of Lemma 6 that it transforms *c* into three components $c'$, $c''$, and $c'''$ such that two of them are odd components and the other has the same parity as $c_r$.

If *c* is odd, and if we can always increase the number of components by two units, we end up with a component with only one intergenic region, but if *c* is even, at some point we will have to increase the number of components by one unit, creating two odd components. This means that for each even component we need to apply two 2-transpositions that increase the number of components by one unit only. Since we can always apply pairs of transpositions that do not increase the number of even components, it follows that $d(\pi ) = m - C(I(\pi ,r^\pi ,r^\iota )) + C_{even}(I(\pi ,r^\pi ,r^\iota ))$. $\square$

Lemmas 8 and 9 respectively show the lower and upper bounds for finding $d(\pi )$ using super short transpositions.

### **Lemma 8**

*Let* $(\pi ,r^\pi )$ *be an instance,* $(\iota ,r^\iota )$ *be the target instance, and let* $m=n+1$ *be the number of intergenic regions in* $r^\pi$ *and* $r^\iota$. *It follows that* $d(\pi ) \ge \max \{\frac{m-C(I(\pi ,r^\pi ,r^\iota ))+C_{even}(I(\pi ,r^\pi ,r^\iota ))}{2},inv(\pi )\}$.

### *Proof*

In order to sort $\pi$ we need to remove all inversions, and since a 2-transposition can remove only one inversion, we necessarily have that $d(\pi ) \ge inv(\pi )$. Besides, by Lemma 2, we can increase the number of components by at most two with a super short transposition. Let $k=C(I(\pi ,r^\pi ,r^\iota ))-C_{even}(I(\pi ,r^\pi ,r^\iota ))$. To reach *m* trivial components, and considering also Lemma 7, we need at least $\frac{m+k}{2}$ super short transpositions. Thus, $d(\pi ) \ge \max \{\frac{m+k}{2},inv(\pi )\}$. $\square$

### **Lemma 9**

*Let* $(\pi ,r^\pi )$ *be an instance,* $(\iota ,r^\iota )$ *be the target instance, and let* $m=n+1$ *be the number of intergenic regions in* $r^\pi$ *and* $r^\iota$. *We have that* $d(\pi ) \le inv(\pi ) + m - C(I(\pi ,r^\pi ,r^\iota )) + C_{even}(I(\pi ,r^\pi ,r^\iota ))$.

### *Proof*

Suppose that we first remove all inversions of $\pi$ using $inv(\pi )$ 2-transpositions of type $\tau (i,i+1,i+2,\ell (r_i^{\pi }),0,0)$, and let $\pi '$ be the resulting permutation. The value of $C(I(\pi ',r^{\pi '},r^\iota ))$ cannot be smaller than $C(I(\pi ,r^{\pi },r^\iota ))$ since any 2-transposition removing an inversion is applied inside a same component. Let $k = C(I(\pi ,r^\pi ,r^\iota ))-C_{even}(I(\pi ,r^\pi ,r^\iota ))$ and let $k' = C(I(\pi ',r^{\pi '},r^\iota ))-C_{even}(I(\pi ',r^{\pi '},r^\iota ))$

Let us analyze the parity of any component that a 2-transposition breaks: (i) if it transforms an odd component into two, then one component must be odd; (ii) if it transforms an even component into two, then both components are odd or even; (iii) if it transforms an even component into three, then two components must be odd; (iv) if it transforms an odd component into three, then either two components are even or the three components are odd. This means that $k' \ge k$.

By Lemma 7, we can go from $C(I(\pi ',r^{\pi '},r^\iota ))$ to *m* components using $m-k'$ 2-transpositions, which results, by the analysis above, in no more than $m-k$ 2-transpositions. $\square$

Finally, using Lemmas 8 and 9, we prove that it is possible to obtain a 3-approximable solution for this problem.

### **Theorem 3**

*Let* $(\pi ,r^\pi )$ *be an instance,* $(\iota ,r^\iota )$ *be the target instance, and let* $m=n+1$ *be the number of intergenic regions in* $r^\pi$ *and* $r^\iota$. *The value of* $d(\pi )$ *is* 3-*approximable.*

### *Proof*

Let $k = C(I(\pi ,r^\pi ,r^\iota ))-C_{even}(I(\pi ,r^\pi ,r^\iota ))$. If $\frac{m-k}{2} \ge inv(\pi )$ then, by Lemma 8, $d(\pi ) \ge \frac{m-k}{2}$, and, by Lemma 5, $d(\pi ) \le m-k+inv(\pi ) \le m-k +\frac{m-k}{2} \le 3 \frac{m-k}{2}$. Otherwise, $\frac{m-k}{2} < inv(\pi )$, so $m-k < 2 \; inv(\pi )$. By Lemma 8, $d(\pi ) \ge inv(\pi )$, and, by Lemma 9, $d(\pi ) \le m-k+inv(\pi ) \le 2 \; inv(\pi ) + inv(\pi ) \le 3 \; inv(\pi )$. $\square$

Algorithm 2 describes a 3-approximation algorithm that transforms an instance $(\pi ,r^\pi )$ into $(\iota ,r^\iota )$ using Super Short Transpositions. Similarly to Algorithm 1, Algorithm 2 has a time complexity of $O(n^2)$.

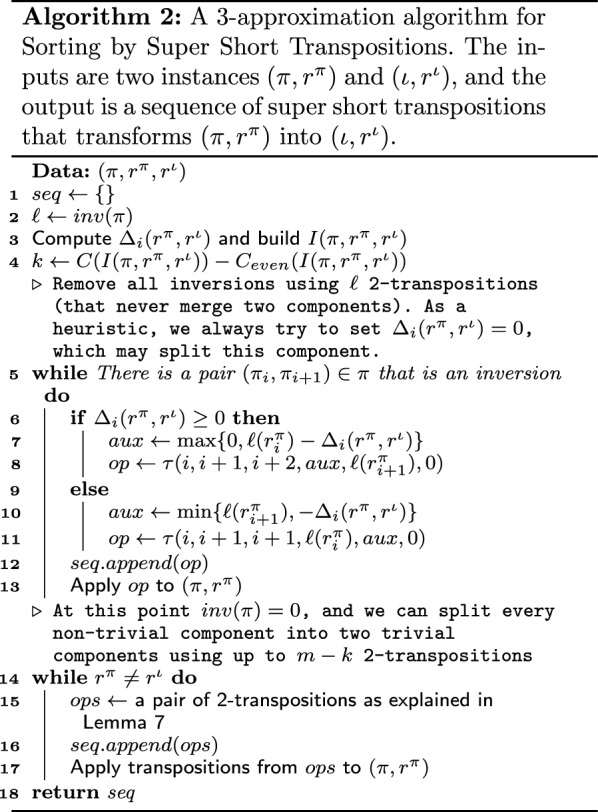



Algorithm 2 has a better approximation factor when the number of inversions is strictly greater than *n*, as stated in the following theorem.

### **Theorem 4**

*Let* $(\pi ,r^\pi )$ *be an instance,* $(\iota ,r^\iota )$ *be the target instance, and let* $m=n+1$ *be the number of intergenic regions in* $r^\pi$ *and* $r^\iota$. *If* $inv(\pi ) > n$, *Algorithm* 2 *has an approximation factor of* $(1+\frac{1}{\ell })$, *where* $\ell = \frac{inv(\pi )}{n} \ge 1$.

### *Proof*

Similar to proof of Theorem 2, given that $m-C(I(\pi ,r^\pi ,r^\iota ))+C_{even}(I(\pi ,r^\pi ,r^\iota )) \le n+1$. $\square$

### Corollary 4.1

*Let* $(\pi ,r^\pi )$ *be an instance,* $(\iota ,r^\iota )$ *be the target instance, and let* $m = n+1$ *be the number of intergenic regions in* $r^\pi$ *and* $r^\iota$. *If* $inv(\pi ) \ge n$ (*resp.* $inv(\pi ) \ge 2n$) *Algorithm* 2 *has an approximation factor of at most 2 (resp. 1.5).*

## Sorting by Super Short Reversals and Super Short Transpositions

In this section we analyze the version of the problem when both super short reversals and Super Short Transpositions are allowed to transform any $(\pi ,r^\pi )$ into $(\iota ,r^\iota )$.

### **Lemma 10**

*Let* $(\pi ,r^\pi )$ *be an instance,* $(\iota ,r^\iota )$ *be the target instance, and let* $m=n+1$ *be the number of intergenic regions in* $r^\pi$ *and* $r^\iota$. *It follows that* $d(\pi ) \ge \max \{\frac{m-C(I(\pi ,r^\pi ,r^\iota ))}{2},inv(\pi )\}$.

### *Proof*

Directly from Lemmas 2, 4, and 8. $\square$

### **Lemma 11**

*Let* $(\pi ,r^\pi )$ *be an instance,* $(\iota ,r^\iota )$ *be the target instance, and let* $m=n+1$ *be the number of intergenic regions in* $r^\pi$ *and* $r^\iota$. *We have that* $d(\pi ) \le inv(\pi ) + m-C(I(\pi ,r^\pi ,r^\iota ))$.

### *Proof*

Suppose that first we remove all inversions of $\pi$ using $inv(\pi )$ 2-reversals of type $\rho (i,i+1,\ell (r_i^{\pi }),0)$, and let $\pi '$ (resp. $r^{\pi '})$) be the resulting permutation (resp. intergenic regions). Let $k = C(I(\pi ,r^{\pi },r^\iota ))$ and let $k' = C(I(\pi ',r^{\pi '},r^\iota ))$. We have that $k' \ge k$, since 2-reversals removing inversions are always applied inside a same component.

Analogous to Lemma 9, and assuming that $k' = k + \ell$ for some $\ell \ge 0$, then $C_{even}(I(\pi ',r^{\pi '},r^\iota )) \le C_{even}(I(\pi ,r^{\pi },r^\iota ))+\ell$. We use the procedure described in Lemma 9 on components *c* with $c_r \ge 3$, applying two 2-transpositions that increase the number of components by two units. For components *c* with $c_r = 2$, we apply a 1-reversal as described in Lemma 1, breaking them into two odd components.

The above procedure applies $inv(\pi )$ 2-reversals, $n - k' - C_{even}(I(\pi ',r^{\pi '},r^\iota ))$ 2-transpositions, and $C_{even}(I(\pi ',r^{\pi '},r^\iota ))$ 1-reversals, which results in no more than $inv(\pi ) + m -C(I(\pi ),r^\iota ))$. $\square$

Now we prove that it is possible to obtain a 3-approximable solution for this problem.

### **Theorem 5**

*Let* $(\pi ,r^\pi )$ *be an instance,* $(\iota ,r^\iota )$ *be the target instance, and let* $m=n+1$ *be the number of intergenic regions in* $r^\pi$ *and* $r^\iota$. *The value of* $d(\pi )$ *is* 3-*approximable.*

### *Proof*

Similar to proof of Theorem 1, using Lemmas 10 and 11. $\square$

Algorithm 3 describes a 3-approximation algorithm that transforms an instance $(\pi ,r^\pi ,r^\iota )$ into $(\iota ,r^\iota ,r^\iota )$ using both super short reversals and super short transpositions. As algorithms 1 and  2, it has a time complexity of $O(n^2)$.

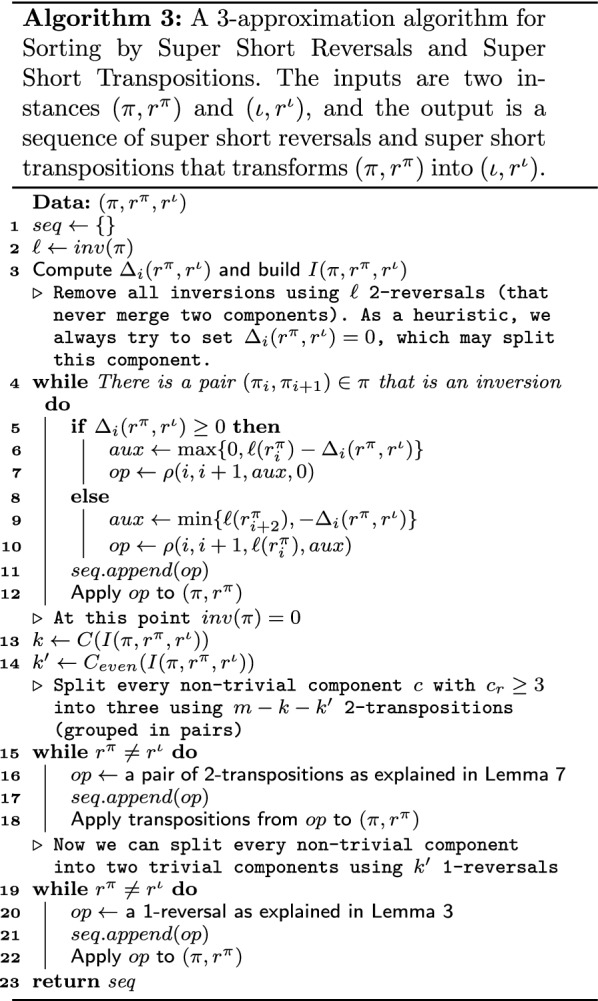



As in the previous algorithms, Algorithm 3 has a better approximation factor when the number of inversions is at least *n*, as explained in the following theorem.

### **Theorem 6**

*Let* $(\pi ,r^\pi )$ *be an instance,* $(\iota ,r^\iota )$ *be the target instance, and let* $m=n+1$ *be the number of intergenic regions in* $r^\pi$ *and* $r^\iota$. *If* $inv(\pi ) \ge n$ *Algorithm* 3 *has an approximation factor of* $(1+\frac{1}{\ell })$, *where* $\ell = \frac{inv(\pi )}{n} \ge 1$.

### *Proof*

Analogous to proof of Theorem 2. $\square$

### Corollary 6.1

*Let* $(\pi ,r^\pi )$ *be an instance,* $(\iota ,r^\iota )$ *be the target instance, and let* $m = n+1$ *be the number of intergenic regions in* $r^\pi$ *and* $r^\iota$. *If* $inv(\pi ) \ge n$ (*resp.* $inv(\pi ) \ge 2n$) *Algorithm* 3 *has an approximation factor of at most 2 (resp. 1.5).*

## Sorting by Signed Super Short Reversals

In this section, we analyze the version of the problem when super short reversals are allowed to transform $(\pi ,r^\pi )$ into $(\iota ,r^\iota )$, where $\pi$ and $\iota$ are signed permutations.

Given a signed permutation $\pi$, let $S^{even^-}_\pi$ be the set of elements from $\pi$ such that $||\pi _i|-i|$ is even and $\pi _i < 0$, and let $S^{odd^+}_\pi$ be the set of elements from $\pi$ such that $||\pi _i|-i|$ is odd and $\pi _i > 0$. Sets $S^{even^-}_\pi$ and $S^{odd^+}_\pi$ capture the negative and positive elements from $\pi$ that end with negative signs after any sequence of 2-reversals that puts all elements in their correct positions (i.e., remove all inversions). Let $\varphi ^{neg}$ be the number of elements in $S^{even^-}_\pi \cup S^{odd^+}_\pi$.

The following lemma, proved by Galvão et al. [8], gives the exact number of super short reversals needed to transform $\pi$ into $\iota$.

### **Lemma 12**

*Given a signed permutation* $\pi$, $d(\pi ) = inv(\pi ) + \varphi ^{neg}$.

This lemma helps us to state the following lower bound for our problem.

### **Lemma 13**

*Let* $(\pi ,r^\pi )$ *be an instance,* $(\iota ,r^\iota )$ *be the target instance, and let* $m=n+1$ *be the number of intergenic regions in* $r^\pi$ *and* $r^\iota$. *We have that* $d(\pi ) \ge inv(\pi ) + \max \{\varphi ^{r},\varphi ^{neg}\}$.

### *Proof*

Directly from Lemmas 4 and 12. $\square$

The following lemma states an upper bound for this problem.

### **Lemma 14**

*Let* $(\pi ,r^\pi )$ *be an instance,* $(\iota ,r^\iota )$ *be the target instance, and let* $m=n+1$ *be the number of intergenic regions in* $r^\pi$ *and* $r^\iota$. *We have that* $d(\pi ) \le inv(\pi ) + \max \{\varphi ^{r},\varphi ^{neg}\} + 2(m-C(I(\pi ,r^\pi ,r^\iota ))$.

### *Proof*

Let $k = C(I(\pi ,r^\pi ,r^\iota ))$ and let $\ell = \max \{\varphi ^{r},$ $\varphi ^{neg}\}$. Suppose that we first remove all inversions of $\pi$ using $inv(\pi )$ 2-reversals of type $\rho (i,i+1,\ell (r_i^{\pi }),0)$, and let $\pi '$ (resp. $r^{\pi '}$) be the resulting permutation (resp. intergenic regions).

Let $k' = C(I(\pi ',r^{\pi '},r^\iota )$. We have that $k' \ge k$. We apply $m-k' \le m-k$ 1-reversals that split every non-trivial component from $I(\pi ',r^{\pi '},r^\iota )$ into two components according to Lemma 3, and let $\pi ''$ (resp. $r^{\pi ''}$) be the resulting permutation (resp. intergenic regions).

At this point, we have a permutation $\pi ''$ such that $r^{\pi ''} = r^\iota$, and $\pi ''$ has no more than $\ell +(m-k') \le \ell +(m-k)$ negative elements. We just need to apply up to $\ell +(m-k')$ 1-reversals of type $\rho (i,i,\ell (r^{\pi ''}_i),0)$ (i.e., without modifying the length of its intergenic regions) to each negative element from $\pi '$, and the lemma follows. $\square$

Using Lemmas 13 and 14, we prove that the value of $d(\pi )$ is 5-approximable.

### **Theorem 7**

*Let* $(\pi ,r^\pi )$ *be an instance,* $(\iota ,r^\iota )$ *be the target instance, and let* $m=n+1$ *be the number of intergenic regions in* $r^\pi$ *and* $r^\iota$. *The value of* $d(\pi )$ *is* 5*-approximable.*

### *Proof*

Let $k = C(I(\pi ,r^\pi ,r^\iota ))$, and let $\ell = \max \{\varphi ^{r},$ $\varphi ^{neg}\}$. If $\frac{m-k}{2} \ge inv(\pi ) + \ell$ then, by Lemma 12, $d(\pi ) \ge \frac{m-k}{2}$, and, by Lemma 14, $d(\pi ) \le 2(m-k)+inv(\pi )+\ell \le 2(m-k) +\frac{m-k}{2} \le 5 \frac{m-k}{2}$.

Otherwise, $\frac{m-k}{2} < inv(\pi ) + \ell$, so $2(m-k) < 4(inv(\pi )+\ell )$. By Lemma 12, $d(\pi ) \ge inv(\pi ) + \ell$, and, by Lemma 14, $d(\pi ) \le 2(m-k)+inv(\pi ) + \ell \le 4(inv(\pi )+\ell ) + inv(\pi ) + \ell \le 5(inv(\pi )+\ell )$. $\square$

Algorithm 4 describes a 5-approximation algorithm that transforms a signed instance $(\pi ,r^\pi ,r^\iota )$ into $(\iota ,r^\iota ,r^\iota )$ using Signed Super Short Reversals. As in previous algorithms, the time complexity of Algorithm 4 is $O(n^2)$.

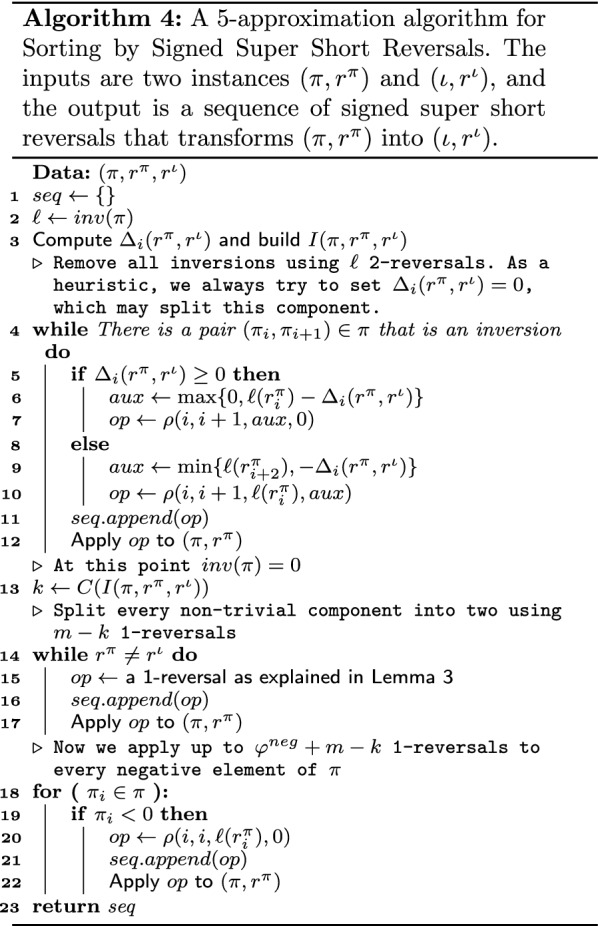



As in the previous algorithms, Algorithm 4 has a better approximation factor when the number of inversions is at least *n*, as explained in the following theorem.

### **Theorem 8**

*Let* $(\pi ,r^\pi )$ *be an instance,* $(\iota ,r^\iota )$ *be the target instance, and let* $m=n+1$ *be the number of intergenic regions in* $r^\pi$ *and* $r^\iota$. *If* $inv(\pi ) \ge n$, *Algorithm* 3 *has an approximation factor of* $(1+\frac{2}{\ell })$, *where* $\ell = \frac{inv(\pi )}{n} \ge 1$.

### *Proof*

Let $k = C(I(\pi ,r^\pi ,r^\iota ))$. Suppose now that $inv(\pi ) = n\ell$ for some $\ell \ge 1$. Since $\frac{m-k}{2} < n$, by Lemma 4 we have that $d(\pi ) \ge n\ell$. Algorithm 4 applies $n\ell$ 2-reversals, up to $m-k < n$ 1-reversals, and up to *n* 1-reversals to flip the sign of each negative element, which results in no more than $n\ell + n-1 + n < n(\ell +2)$ super short reversals. $\square$

### Corollary 8.1

*Let* $(\pi ,r^\pi )$ *be an instance,* $(\iota ,r^\iota )$ *be the target instance, and let* $m = n+1$ *be the number of intergenic regions in* $r^\pi$ *and* $r^\iota$. *If* $inv(\pi ) \ge n$ (*resp.* $inv(\pi ) \ge 2n$) *Algorithm* 4 *has an approximation factor of at most 3 (resp. 2).*

## Sorting by Signed Super Short Reversals and Super Short Transpositions

In this section, we analyze the version of the problem when both super short reversals and Super Short Transpositions are allowed to sort signed permutations.

Let $H(\pi )$ be the *inversion graph* [18] of the signed permutation $\pi$, such that $V(H(\pi )) = \{\pi _1,\pi _2,...,\pi _n\}$ and $E(H(\pi ))$ is formed by pairs of elements from $\pi$ that are inversions. In $H(\pi )$, a component is defined as a maximal subgraph in which any two vertices are connected to each other by paths. A component from $H(\pi )$ is *negative* if it contains an odd number of negative elements (vertices), and it is *positive* otherwise.

Let $\varphi ^{odd}$ be the number of negative components of $H(\pi )$. The following lemma, proved by Galvão et al. [8], gives the exact number of super short reversals and Super Short Transpositions needed to transform $\pi$ into $\iota$, which is a lower bound for our problem.

### **Lemma 15**

*Given a signed permutation* $\pi$, $inv(\pi ) + \varphi ^{odd}$ *super short operations are required to transform* $\pi$ *into* $\iota$.

Now we state in the following lemma an upper bound for this problem.

### **Lemma 16**

*Let* $(\pi ,r^\pi )$ *be an instance,* $(\iota ,r^\iota )$ *be the target instance, and let* $m=n+1$ *be the number of intergenic regions in* $r^\pi$ *and* $r^\iota$. *We have that* $d(\pi ) \le inv(\pi ) + \varphi ^{odd} + 2(m-C(I(\pi ,r^\pi ,r^\iota )))$.

### *Proof*

Suppose that we first remove all inversions of $\pi$ using the polynomial algorithm presented in [8], that uses $inv(\pi ) + \varphi ^{odd}$ super short operations such that all 2-reversals are of type $\rho (i,i+1,\ell (r^\pi _i),0)$, all 2-transpositions are of type $\tau (i,i+1,i+2,\ell (r^\pi _i),0,0)$, and all the $\varphi^{odd}$ 1-reversals are ignored (i.e., not applied),  and let $\pi '$ be the resulting permutation.

The number of components $I(\pi ',r^{\pi '},r^\iota )$ in $\pi '$ cannot be smaller than $C(I(\pi ,r^\pi ,r^\iota ))$, since the 2-reversals and 2-transpositions are applied inside a same component only. Let $k' = C(I(\pi ',r^{\pi '},r^\iota )) \ge C(I(\pi ,r^\pi ,r^\iota ))$.

By Lemma 3, we can go from $k'$ to *m* components using $m-{k'}$ 1-reversals, which results in no more than $m{-C(I(\pi ,r^\pi ,r^\iota ))}$ 1-reversals. After that, we will have a permutation $\pi ''$ with up to $\min\{n,m-k'+\varphi^{odd}\}$ negative elements, so we can apply up to $\min\{n,m-k'+\varphi^{odd}\}$ 1-reversals of type $\rho (i,i,\ell (r^\iota _i),0)$ to each negative element of $\pi ''$. $\square$

Using Lemmas 15 and 16, we prove that it is possible to obtain a 5-approximable solution for this problem.

### **Theorem 9**

*Let* $(\pi ,r^\pi )$ *be an instance,* $(\iota ,r^\iota )$ *be the target instance, and let* $m=n+1$ *be the number of intergenic regions in* $r^\pi$ *and* $r^\iota$. *The value of* $d(\pi )$ *is* 5*-approximable.*

### *Proof*

Let $k = C(I(\pi ,r^\pi ,r^\iota ))$, and let $\ell = \varphi ^{odd}$. If $\frac{m-k}{2} \ge inv(\pi ) + \ell$ then, by Lemma 15, $d(\pi ) \ge \frac{m-k}{2}$, and, by Lemma 16, $d(\pi ) \le 2(m-k)+inv(\pi )+\ell \le 2(m-k) +\frac{m-k}{2} \le 5 \frac{m-k}{2}$.

Otherwise, $\frac{m-k}{2} < inv(\pi ) + \ell$, so $2(m-k) < 4(inv(\pi )+\ell )$. By Lemma 15, $d(\pi ) \ge inv(\pi ) + \ell$, and, by Lemma 16, $d(\pi ) \le 2(m-k)+inv(\pi ) + \ell \le 4(inv(\pi )+\ell ) + inv(\pi ) + \ell \le 5(inv(\pi )+\ell )$. $\square$

Algorithm 5 describes a 5-approximation algorithm that transforms a signed instance $(\pi ,r^\pi ,r^\iota )$ into $(\iota ,r^\iota ,r^\iota )$ using both signed super short reversals and Super Short Transpositions. Regarding the complexity, by previous algorithms we know that lines 1-3 and 6-17 take up to $O(n^2)$, and according to [8] the while loop in line 4 takes $O(n^3)$, which is then the time complexity of Algorithm 5.

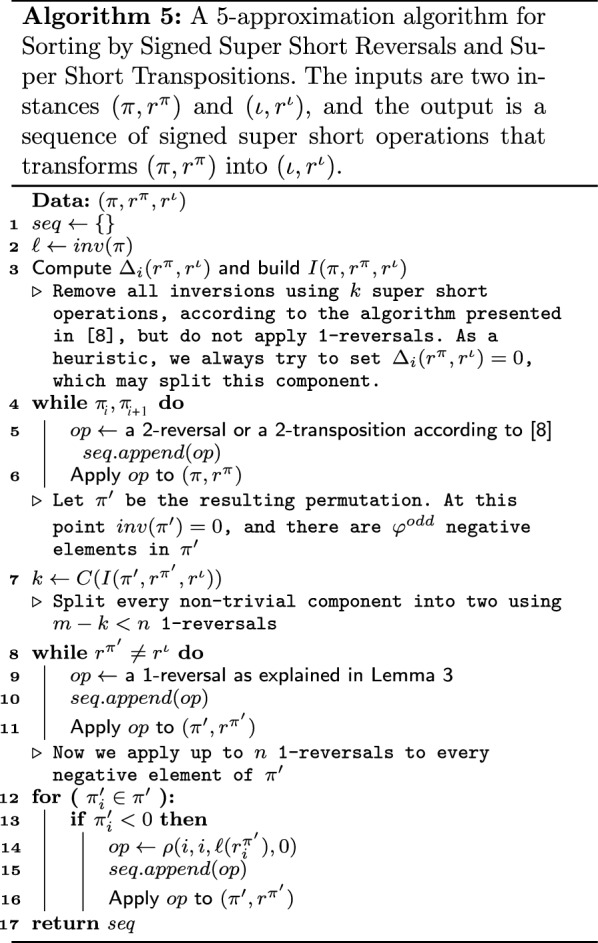



As for previous algorithms, Algorithm 5 also has a better approximation factor when the number of inversions is at least *n*. This is the purpose of the following theorem.

### **Theorem 10**

*Let* $(\pi ,r^\pi )$ *be an instance,* $(\iota ,r^\iota )$ *be the target instance, and let* $m=n+1$ *be the number of intergenic regions in* $r^\pi$ *and* $r^\iota$. *If* $inv(\pi ) \ge n$, *Algorithm* 5 *has an approximation factor of* $(1+\frac{2}{\ell })$, *where* $\ell = \frac{inv(\pi )}{n} \ge 1$.

### *Proof*

Let $k = C(I(\pi ,r^\pi ,r^\iota ))$. Suppose now that $inv(\pi ) = n\ell$. Since $\frac{m-k}{2} < n$, by Lemma 4 we have that $d(\pi ) \ge \ell$. Algorithm 4 applies $n\ell$ operations between 2-reversals and 2-transpositions, up to $m-k < n$ 1-reversals, and up to *n* 1-reversals to flip the sign of each negative element, which results in no more than $n\ell + n-1 + n < n(\ell +2)$ super short operations. $\square$

### Corollary 10.1

*Let* $(\pi ,r^\pi )$ *be an instance,* $(\iota ,r^\iota )$ *be the target instance, and let* $m = n+1$ *be the number of intergenic regions in* $r^\pi$ *and* $r^\iota$. *If* $inv(\pi ) \ge n$ (*resp.* $inv(\pi ) \ge 2n$) *Algorithm* 5 *has an approximation factor of at most 3 (resp. 2).*

## Experimental tests

We implemented the five proposed algorithms and tested them unsing simulated permutations, in order to observe their performances. We generated two different permutation datasets, which we call fully-random instances (FRI) and almost random instances (ARI). Each dataset has 1,000,000 instances $(\pi ,r^\pi )$, $\pi$ is a permutation with 100 elements and $r^\pi$ is a sequence of 101 intergenic regions sizes.

The dataset FRI was generated in the following way: (i) let $(\iota ,r^\iota )$ be an initial instance, being $\iota$ with 100 elements, and each $r^\iota _i$ received a random integer $k \in [0..100]$. (ii) Generate $(\pi ,r^\pi )$ by applying *w* consecutive super short operations to $(\iota ,r^\iota )$, with randomly generated indices for both positions and intergenic sizes, always respecting the current values. We created 10,000 instances for each value of $w \in \{10,20,30,\ldots ,990,1000\}.$

For Sorting by (Signed) Super Short Reversals we applied 0.8*w* 2-reversals and 0.2*w* 1-reversals, and at each step one of them was chosen at random while both were available. For Sorting by Super Short Transpositions we applied *w* 2-transpositions. For Sorting by (Signed) Super Short Operations we applied 0.5*w* 2-transpositions, 0.4*w* 2-reversals, and 0.1*w* 1-reversals, and at each step one of them was chosen at random while more than one were available.

The dataset ARI was generated in a similar way as FRI, but when the algorithm had to apply either a 2-reversal or a 2-transposition we randomly chose a pair among all pairs of adjacent elements that were not an inversion. Since $w < \max \{inv(\pi ), \pi \in \delta_n\} = \frac{n(n-1)}{2}$, at least one pair always exists.

Given any instance $(\pi ,r^\pi )$ from ARI created using *w* SSOs, we know exactly how many inversions $(\pi ,r^\pi )$ has—it is the number of 2-reversals and 2-transpositions applied. The number of inversions on instances from FRI, however, is not known, but we can compute the expected number of inversions in a permutation with *n* elements after *k* random swaps (i.e., 2-reversals and 2-transpositions) applied to the identity permutation [19]:

$$\begin{aligned} E[i_{n,k}] = \frac{n(n+1)}{4} - \frac{1}{8(n+1)^2} \sum \limits _{i,j=0}^{n} \frac{(c_j+c_i)^2}{s_k^2s_i^2} x_{ji}^k, \end{aligned}$$

where $$c_a = \cos {\alpha _a}$$, $$s_a = \sin {\alpha _a}$$, $$x_{ab} = 1 - \frac{4}{n}(1-c_ac_b)$$, and $$\alpha _a = \frac{(2a+1)\pi }{2n+2}$$.

**Fig. 5:**
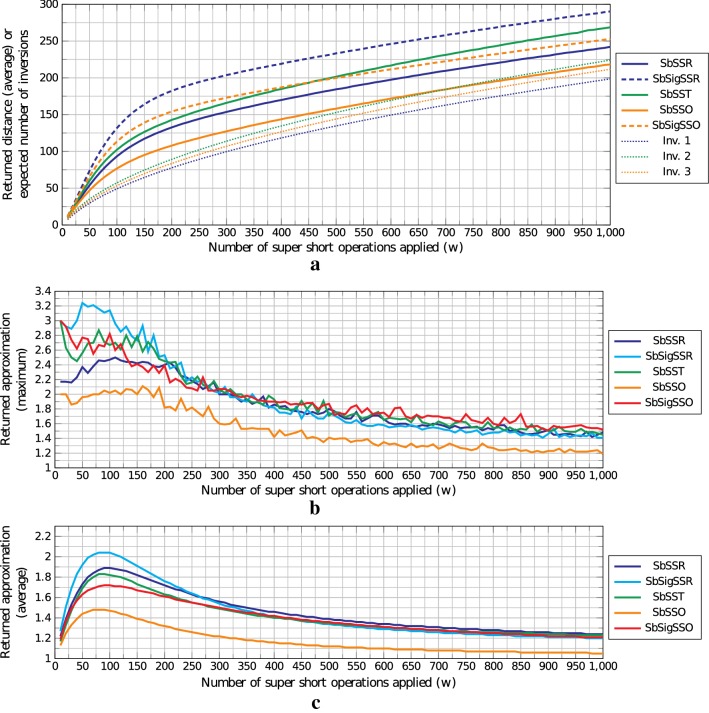
**a** average returned distance, **b** maximum returned approximation, and **c** average returned approximation for instances in FRI. In **a** dotted curves represent the expected number of inversions, dashed curves represent the algorithms for signed permutations, and line curves represent the algorithms for unsigned permutations. Colors relate problems having the expected number of inversions given by the dotted line of same color. This means that SbSSR and SbSigSSR are expected to have inversions as in Inv. 1, SbSST is expected to have inversions as in Inv. 2, and SbSSO and SbSigSSO are expected to have inversions as in Inv. 3. All the approximations in **b** and **c** were calculated based on the lower bound of each problem

**Fig. 6:**
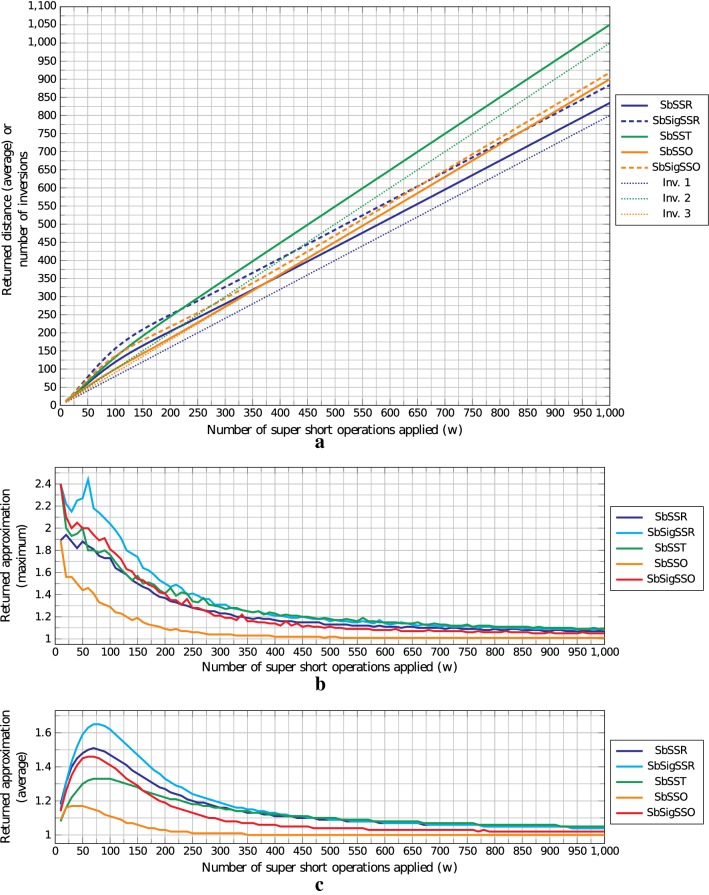
**a** average returned distance, **b** maximum returned approximation, and **c** average returned approximation for instances in ARI. In **a** dotted curves represent the exact number of inversions, dashed curves represent the algorithms for signed permutations, and line curves represent the algorithms for unsigned permutations. Colors relate problems having the expected number of inversions given by the dotted line of same color. This means that SbSSR and SbSigSSR have inversions as in Inv. 1, SbSST has inversions as in Inv. 2, and SbSSO and SbSigSSO have inversions as in Inv. 3. All the approximations in **b** and **c** were calculated based on the lower bound of each problem

Figures 5 and 6 show the experimental results for instances of type FRI and ARI, respectively. We show the average distance returned for each algorithm described in this paper, plus the average and maximum approximation factors calculated based on the lower bound of each problem for each instance.

On Figs. 5 and 6, Algorithm 1 is denoted by SbSSR, Algorithm 4 is denoted by SbSigSSR, Algorithm 2 is denoted by SbSST, Algorithm 3 is denoted by SbSSO, and Algorithm 5 is denoted by SbSigSSO. In Fig. 5, the curve Inv. 1 represents the expected number of inversions for SbSSR and SbSigSSR, the curve Inv. 2 represents the expected number of inversions for SbSST, and the curve Inv. 3 denotes the expected number of inversions for SbSSO and SbSigSSO. These three curves were generated using the formula $E[i_{n,k}]$ described above. In Fig. 6 the curves Inv. 1, 2, and 3 follow the same idea as the curves in Fig. 5, but instead of expected number of inversions they represent the exact number of inversions.

As distance is directly related to the number of inversions, in Fig. 5a we see that, although in practice we have applied up to 1000 operations, the distance values were never greater than 300 on average—the average returned distances for each algorithm follow the trend dotted line that represents the expected number of inversions of that instances. Algorithms for signed permutations returned distances with a slightly higher value than the same algorithms for unsigned permutations, which is expected given that in addition to inversions and intergenic sizes, they also need to take care of elements with negative signs.

Concerning the approximation factors in Fig. 5b, c, it can be noted that despite the theoretical approximation factors of 3 and 5, the average approximation factors of instances in FRI were between 1 and 2.2. Furthermore, in our tests, no instance for SbSSR and SbSSO, whose theoretical approximation factors are 3, had approximation factor above 2.5, and no instance for SbSigSSR and SbSigSSO, whose theoretical approximation factors are 5, obtained approximation above 3.3 and 3, respectively.

In Fig. 6a we have another scenario where the returned distance follows the number of applied SSOs, but this behavior is due to our choice of applying only operations that do not destroy previously created inversions. One interesting thing about this figure is that distances returned by the algorithm for SSOs were very close to the number of inversions, especially when $w \ge 400$, something that did not happened on FRI. In Fig. 6b, c, we see that this dataset returned maximum and average approximations systematically better than for dataset FRI: no instance had an approximation factor greater than 2.5, and on average all algorithms have average approximation factors less than 1.7. For $w \ge 120$ (resp. $w \ge 240$), where we expect to have around *n* (resp. 2*n*) inversions, none of the instances had approximation factor above 2 (resp. 1.5), as we expected given Lemmas 2, 4, 6, 8, and 10.

## Conclusion

In this paper, we analyzed the minimum number of super short reversals and/or Super Short Transpositions needed to sort a signed or unsigned permutation $\pi$ and, at the same time, transform its intergenic regions lengths $r^\pi$ according to $r^\iota$.

We defined some bounds and a graph structure that allowed us to build five algorithms (one for each considered problem) that guarantee approximation factors of 3 for unsigned permutations (using either SSRs, SSTs, or both) and 5 for signed permutations (using either SSRs or SSOs). These algorithms have better approximation factors for instances for which the number of inversions is at least *n* or 2*n*. In the former case, it is equal to 2 for unsigned permutations (using either SSRs, SSTs, or both), and to 3 for signed permutations (using SSRs or SSOs); in the latter case it is equal to 1.5 for unsigned permutations, and to 2 for signed permutations. All of these algorithms were tested in simulated instances, showing that, on average, they behave better than their theoretical approximation factors predict.

Some questions remain open. For instance, what is the computational complexity of each of these five problems? Besides, how can we incorporate indels (insertions and deletions) of intergenic regions to these problems, to be able to compare two genomes that share the same set of genes but may differ on their total intergenic regions length?
